# Sargachromenol from *Sargassum micracanthum* Inhibits the Lipopolysaccharide-Induced Production of Inflammatory Mediators in RAW 264.7 Macrophages

**DOI:** 10.1155/2013/712303

**Published:** 2013-09-30

**Authors:** Eun-Jin Yang, Young Min Ham, Kyong-Wol Yang, Nam Ho Lee, Chang-Gu Hyun

**Affiliations:** ^1^Jeju Biodiversity Research Institute (JBRI), Jeju Technopark, Jeju 699-943, Republic of Korea; ^2^Jeju Love Co., Ltd., 542-5 Haengwon-ri, Gujwa-eup, Jeju 695-975, Republic of Korea; ^3^Cosmetic Science Center, Department of Chemistry, Jeju National University, Jeju 690-756, Republic of Korea; ^4^LINC Agency, Jeju National University, Ara-1-dong, Jeju 690-756, Republic of Korea

## Abstract

During our ongoing screening program designed to determine the anti-inflammatory potential of natural compounds, we isolated sargachromenol from *Sargassum micracanthum*. In the present study, we investigated the anti-inflammatory effects of sargachromenol on lipopolysaccharide (LPS)-induced inflammation in murine RAW 264.7 macrophage cells and the underlying mechanisms. Sargachromenol significantly inhibited the LPS-induced production of nitric oxide (NO) and prostaglandin E_2_ (PGE_2_) in a dose-dependent manner. It also significantly inhibited the protein expression of inducible NO synthase (iNOS) and cyclooxygenase-2 (COX-2) in a dose-dependent manner in LPS-stimulated macrophage cells. Further analyses showed that sargachromenol decreased the cytoplasmic loss of inhibitor **κ**B**α** (I**κ**B**α**) protein. These results suggest that sargachromenol may exert its anti-inflammatory effects on LPS-stimulated macrophage cells by inhibiting the activation of the NF-**κ**B signaling pathway. In conclusion, to our knowledge, this is the first study to show that sargachromenol isolated from *S. micracanthum* has an effective anti-inflammatory activity. Therefore, sargachromenol might be useful for cosmetic, food, or medical applications requiring anti-inflammatory properties.

## 1. Introduction

Inflammation is the result of the host's immune response to pathogenic challenges or tissue injuries. Once the response ends, tissue structure and function recover their normal states. Moreover, normal inflammatory responses are tightly controlled and self-limiting, with downregulation of proinflammatory proteins and upregulation of anti-inflammatory proteins. Thus, acute inflammation is essentially a beneficial process, particularly that which develops in response to infectious pathogens. Chronic inflammation, however, is an undesirable phenomenon and is involved in the pathogenesis of chronic diseases such as cancer, arthritis, autoimmune disorders, and vascular diseases [1–3]. For these reasons, research has focused on the control of inflammatory reactions. One approach has been the investigation of dietary phytochemicals capable of suppressing inflammation. Many attempts have been made to derive new anti-inflammatory agents from natural sources of phytochemicals that have been considered safe, less toxic, and readily available, although their modes of action mostly remain unclear. Thus, elucidating the molecular mechanisms underlying the anti-inflammatory actions of naturally occurring phytochemicals might be a good strategy for identifying new therapeutic agents [4, 5].

Many studies of seaweed-derived anti-inflammatory compounds have investigated the potential inhibitory effects of natural compounds in an in vitro system, that is, lipopolysaccharide (LPS)-stimulated macrophages [6–9]. Using this system, LPS from gram-negative bacteria has become one of the best characterized stimuli for induction of the upregulation of proinflammatory proteins such as cyclooxygenase-2 (COX-2) and inducible nitric oxide synthase (iNOS). Inflammation is induced by many factors, including proinflammatory enzymes such as NO and PGE_2_, which are indicators of inflammatory activity. NO is involved in inflammation and autoimmune diseases, and its levels are elevated during inflammatory responses. COX-2 catalyzes the conversion of arachidonic acid to prostaglandins and is induced by proinflammatory cytokines or LPS. In addition, the COX-2 and iNOS expression is regulated by activation of NF-*κ*B, a transcription factor. Therefore, iNOS and COX-2 are important anti-inflammatory targets [10–13].

Numerous studies have focused on natural compounds or extracts for improving human health, owing to their safety and low toxicity. Although many phytochemicals derived from terrestrial plants have shown anti-inflammatory effects, only a few studies have focused on the molecular mechanisms underlying the anti-inflammatory actions of phytochemicals and extracts from marine algae such as *Ecklonia cava, Sargassum muticum*, and *S. micracanthum* [5].

*S. micracanthum* is a brown marine alga distributed worldwide, from temperate to subtropical regions. A number of compounds isolated from *S. micracanthum*, including sargaquinoic acid, sargachromenol, and sargassumol, have various pharmacological properties, including antioxidant and antiviral activities [14–17]. During our ongoing screening program designed to identify the anti-inflammatory potential of natural compounds, we isolated fucosterol, sargaquinoic acid, and sargachromenol from *S. micracanthum* by using activity-directed fractionation and characterized their structures using spectroscopy (^1^H and ^13^NMR, IR, and MS) as described previously [15]. However, the biological activities or modes of action of these compounds have not been reported previously, although another Korean group recently reported that sargaquinoic acid has anti-inflammatory activity [18]. Therefore, the present study investigated whether sargachromenol inhibited LPS-induced production of NO and PGE_2_, or the expression of iNOS and COX-2 proteins, through the inhibition of I*κ*B-*α* in macrophages.

## 2. Materials and Methods

### 2.1. Chemicals and Reagents

Sargachromenol was isolated from *S. micracanthum*, essentially as previously reported [15]. LPS, derived from *Escherichia coli*, and dimethylsulfoxide (DMSO) were obtained from Sigma-Aldrich (St. Louis, MO, USA). Dulbecco's modified Eagle's medium (DMEM), fetal bovine serum (FBS), penicillin, and streptomycin were obtained from Invitrogen-Gibco (Grand Island, NY, USA). The antibodies (Abs) used were as follows: anti-iNOS rabbit polyclonal, anti-COX-2 monoclonal Ab (mAb), and anti-inhibitor of NF-*κ*B (I*κ*B*α*) mAb (all purchased from Cell Signaling Technology; Beverly, MA, USA). All other reagents were purchased from Sigma-Aldrich Chemical Co. (St. Louis, MO, USA).

### 2.2. Cell Culture and Cell Viability Assay

RAW 264.7 murine macrophages obtained from the Korean Cell Bank (Seoul, Korea) were cultured in DMEM containing 10% FBS, 100 U/mL penicillin, and 100 *μ*g/mL streptomycin at 37°C in 5% CO_2_. Cell viability was measured by 3-(4,5-dimethylthiazol-2-yl)-2,5-diphenyltetrazolium bromide (MTT) assay. RAW 264.7 cells were cultured in 96-well plates for 18 h, followed by treatment with LPS (1 *μ*g/mL) in the presence of various concentrations of sargachromenol for 24 h. MTT was then added to the medium for 4 h. Finally, the supernatant was removed, and the formazan crystals were dissolved in DMSO. Absorbance was measured at 540 nm. The percentage of cells showing cytotoxicity relative to the control group was determined.

### 2.3. Measurement of Nitrite and Prostaglandin E_2_

RAW 264.7 cells were plated at 1.8 × 10^5^ cells/well in 24-well plates and then incubated with or without LPS (1 *μ*g/mL) in the absence or presence of sargachromenol (12.5, 25, 50, and 100 *μ*M) for 24 h. The NO determination was carried out as described previously [14] by using the Griess reaction. Briefly, conditioned cell culture media (100 *μ*L) were mixed with 100 *μ*L of the Griess reagent (1% sulphanilamide and 0.1% *N*-[1-naphthyl]-ethylenediamine dihydrochloride in 5% phosphoric acid) for 10 min. Then, absorbance was measured at 540 nm using a spectrophotometer. Fresh culture media were used as blanks in all experiments. NO levels in the samples were calculated from a standard curve constructed using sodium nitrite. Sandwich enzyme-linked immunosorbent assay (ELISA) was used to determine the production of cytokines and prostaglandin E_2_ (PGE_2_) in the LPS-treated RAW 264.7 cells in the presence of sargachromenol (12.5, 25, 50, and 100 *μ*M). The RAW 264.7 cells were stimulated by LPS for 24 h before the supernatant was harvested and assayed using the relevant ELISA kit in accordance with the manufacturer's instructions (R&D Systems Inc., Minneapolis, MN, USA). Results from 3 independent experiments were used for statistical analysis.

### 2.4. Western Blotting Analysis

RAW 264.7 cells (1.0 × 10^6^ cells/mL) were preincubated for 18 h and then treated with LPS (1 *μ*g/mL) plus aliquot samples for 24 h. After incubation, the cells were washed twice with 10 mM PBS (pH 7.4) containing 150 mM NaCl and then lysed with RIPA lysis buffer in the presence of protease inhibitors. Whole-cell lysates (30 *μ*g) were separated by 10% sodium dodecyl sulfate-polyacrylamide gel electrophoresis (SDS-PAGE) and electro-transferred to a polyvinylidene fluoride (PVDF) membrane (BIO-RAD, HC). The membrane was incubated for 24 h with 5% skim, milk and then incubated with anti-iNOS, COX-2, I*κ*B*α*, or phosphorylated I*κ*B*α* antibodies (1 : 2500) at room temperature for 2 h. The membrane was washed 4 times with TTBS and then incubated for 30 min with a peroxidase-conjugated secondary antibody (1 : 5000) at room temperature. Finally, the protein bands were visualized using an enhanced chemiluminescence system. The densities of the bands were measured with the ImageQuant LAS 4000 luminescent image analyzer and ImageQuant TL software (GE Healthcare, Little Chalfont, UK).

### 2.5. Statistical Analysis

Results are presented as the mean ± standard deviation of at least 3 replicates. Student's *t*-test was used for statistical analyses of the differences noted. *P* values of 0.05 or less were considered statistically significant.

## 3. Results and Discussion

**Figure 1:**
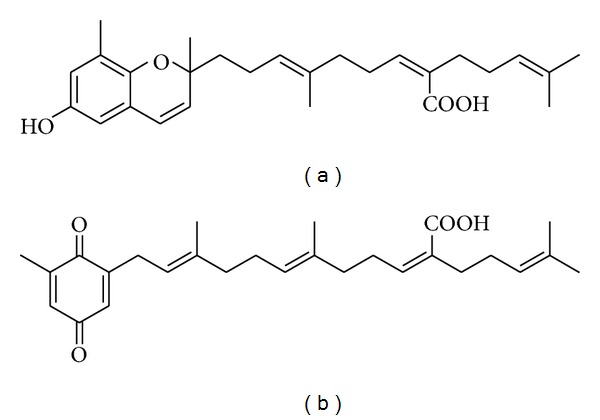
Structures of sargachromenol (a) and sargaquinoic acid (b).

In the course of investigations on the biologically active metabolites from *S. micracanthum* [14], three known compounds, sargaquinoic acid, sargachromenol, and fucosterol, were isolated as major constituents in our previous study (Figure 1). Also, we evaluated their radical scavenging activity against 1,1-diphenyl-2-picrylhydrazyl (DPPH) and hydroxyl radicals using an electron spin trapping technique [15]. Therefore, the present study was undertaken to elucidate the pharmacological and biological effects of sargachromenol on the production of inflammatory mediators in macrophages.

Overproduction of NO, an inflammatory mediator involved in host defense mechanisms, is involved in the pathogenesis of several diseases, including periodontitis, bacterial sepsis, atherosclerosis, bowel disease, rheumatoid arthritis, and septic shock. Pharmacological manipulation of NO production has therefore been speculated to be useful in the alleviation of numerous disease states mediated by increased and/or protracted activation of macrophages [12, 19–21]. PGE_2_, which is produced at inflammatory sites by COX-2, has also been implicated as an important inflammatory mediator. Interestingly, the induction of COX-2 activity and the subsequent generation of PGE_2_ are closely related to NO production. Thus, inhibition of PGE_2_ production is an important therapeutic target in the development of anti-inflammatory agents [22, 23].

**Figure 2:**
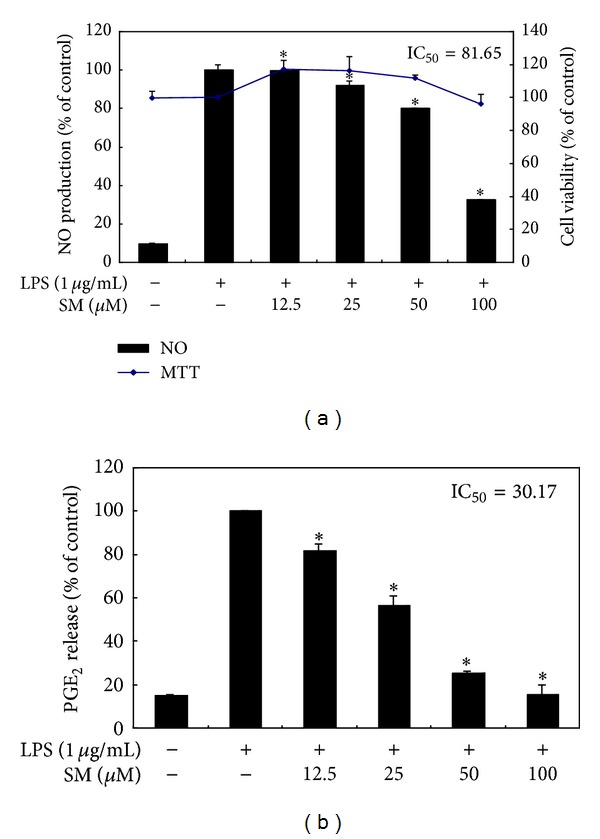
Effect of sargachromenol on NO production in LPS-stimulated RAW 264.7 cells. Cells were stimulated with 1 *μ*g/mL of LPS only or with LPS plus the indicated concentrations of SM for 24 h. NO (a) levels were determined by the Griess reagent method. PGE_2_ (b) levels were measured using an ELISA kit. The data represent the mean ± SD of triplicate experiments. *P < 0.05, **P < 0.01 versus LPS alone. Cell viability was determined from the 24 h culture of cells stimulated with LPS (1 *μ*g/mL) in the presence of SM. NO: nitric oxide; LPS: lipopolysaccharide; SM: sargachromenol; PGE_2_: prostaglandin E_2_; ELISA: enzyme-linked immunosorbent assay.

In order to determine the potential anti-inflammatory properties of sargachromenol on LPS-induced NO/PGE_2_ production, RAW 264.7 cells were treated with sargachromenol (12.5, 25, 50, and 100 *μ*M) or left untreated for 1 h, followed by treatment with LPS (1 *μ*g/mL) for 24 h. NO and PGE_2_ concentrations were measured in the cell culture media via the Griess reaction and ELISA assays, respectively. LPS treatment significantly increased the concentrations of NO and PGE_2_ in the conditioned media. As shown in Figure 2(a), sargachromenol inhibited LPS-induced NO production in a concentration-dependent manner (by 7.7%, 16.8%, 31.0%, and 61.6% in the presence of 12.5, 25, 50, and 100 *μ*M sargachromenol, resp.). Moreover, sargachromenol markedly suppressed LPS-induced PGE_2_ production, with an IC_50_ of 30.2 *μ*M (Figure 2(b)). The cytotoxic effects of sargachromenol were assessed in the presence or absence of LPS by using an MTT assay. As shown in Figure 2(a), sargachromenol did not affect the viability of RAW 264.7 cells at the concentrations employed in this study. Thus, the inhibitory effects on NO and PGE_2_ production were not attributable to cytotoxic effects.

**Figure 3:**
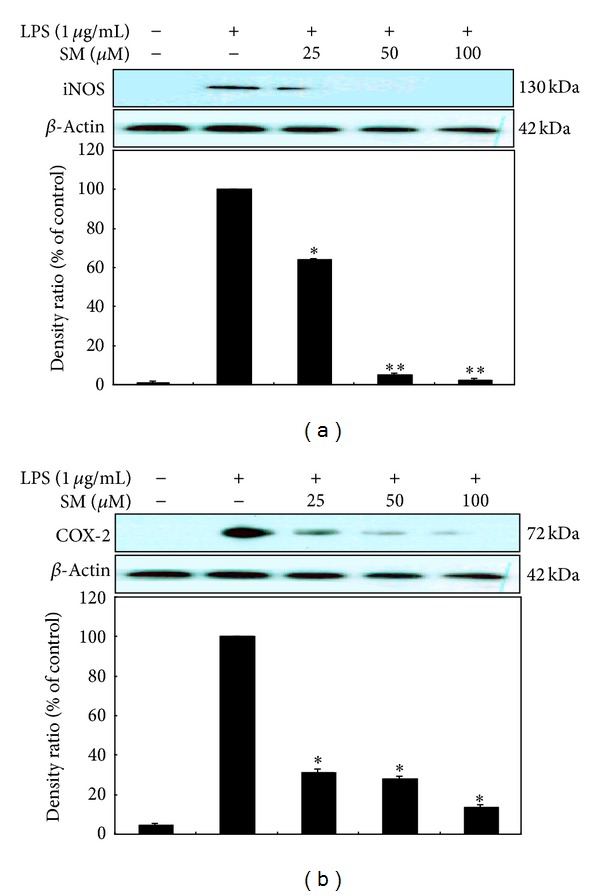
The effect of sargachromenol on the activation of iNOS (a) and COX-2 (b) in LPS-stimulated RAW 264.7 cells. RAW 264.7 cells (5.0 × 10^5^ cells/mL) were stimulated with LPS (1 *μ*g/mL) in the presence of SM at the indicated concentrations for 24 h. Whole-cell lysates (25 *μ*g) were prepared and subjected to 10% SDS-PAGE prior to the analysis of iNOS and *β*-actin (loading control) expression by Western blotting. iNOS: inducible nitric oxide synthase; COX-2: cyclooxygenase-2; LPS: lipopolysaccharide; SM: sargachromenol; SDS-PAGE: sodium dodecyl sulfate-polyacrylamide gel electrophoresis.

In an effort to characterize the anti-inflammatory activities of sargachromenol, we assessed the effects of sargachromenol on LPS-induced iNOS and COX-2 protein upregulation in RAW 264.7 cells by using Western blotting. The levels of iNOS and COX-2 proteins were greatly increased by LPS treatment. However, sargachromenol was found to inhibit the induction of iNOS protein expression in a dose-dependent manner. A densitometric analysis of 3 different experiments demonstrated that LPS-induced iNOS protein expression was inhibited by 94.9% in the presence of 50 *μ*M sargachromenol (Figure 3(a)). In parallel, the LPS-induced expression of COX-2 protein was reduced in the presence of sargachromenol in a concentration-dependent manner (Figure 3(b)). These results led us to evaluate the effects of sargachromenol on the expression of the iNOS and COX-2 enzymes in greater detail.

**Figure 4:**
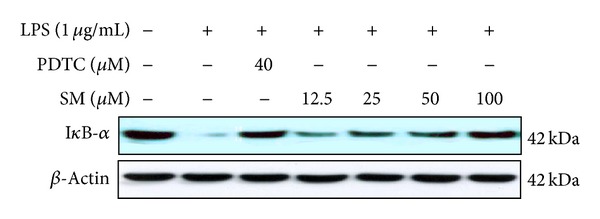
The effects of sargachromenol on the degradation of I*κ*B*α* in LPS-stimulated RAW 264.7 cells. RAW 264.7 cells (1.0 × 10^6^ cells/mL) were stimulated with LPS (1 *μ*g/mL) in the presence of the indicated concentrations of SM or PDTC (40 *μ*M) for 15 min. Whole-cell lysates (30 *μ*g) were prepared and subjected to 12% SDS-PAGE prior to analysis of I*κ*B*α* and *β*-actin (loading control) expression by western blotting. LPS: lipopolysaccharide; SM: sargachromenol; SDS-PAGE: sodium dodecyl sulfate-polyacrylamide gel electrophoresis; PDTC: pyrrolidine dithiocarbamate.

NF-*κ*B is one of the most important transcription factors involved in the transactivation of a variety of genes associated with the regulation of immune and inflammatory responses, including iNOS and COX-2. Activation of NF-*κ*B is correlated with a variety of inflammatory diseases, and many anti-inflammatory drugs inhibit the production of proinflammatory mediators via suppression of the activation of NF-*κ*B suppressors [4–9]. NF-*κ*B is normally bound to an inhibitory subunit, I*κ*B, which is present in the cytoplasm in an inactive form. Foreign stimuli (e.g., LPS or pathogens) induce activation of NF-*κ*B by the phosphorylation, ubiquitination, and subsequent proteolytic degradation of I*κ*B via activated I*κ*B kinase (IKK). The liberated NF-*κ*B transcription factor then translocates to the nucleus and binds to *κ*B motifs in the promoters of target genes, such as those encoding iNOS, COX-2, and cytokines, to promote their transcription [24, 25]. Yoon et al. [26] showed that *Artemisia fukudo* essential oil prevented the degradation of I*κ*B*α* induced by diverse stimuli, and thus, interfered with a step in the signaling cascade leading to the activation of NF-*κ*B. Hyun et al. [27] demonstrated that the anti-inflammatory effects of *Acanthopanax koreanum* fruit waste in RAW 264.7 cells involved prevention of I*κ*B degradation. Therefore, we studied whether sargachromenol inhibited the degradation of I*κ*B*α*. As a control, we also exposed cells to pyrrolidine dithiocarbamate (PDTC), a specific NF-*κ*B inhibitor. As shown in Figure 4, LPS induced transient degradation of I*κ*B in RAW 264.7 cells, whereas sargachromenol and PDTC prevented degradation of I*κ*B in a dose-dependent manner. These results suggested that the inhibition of COX-2 and iNOS expression by sargachromenol occurred via suppression of I*κ*B degradation, thereby preventing NF-*κ*B activation.

In conclusion, the results of the present study provide the first evidence that sargachromenol isolated from *S. micracanthum* inhibits inflammatory mediators, including NO and PGE_2_, in LPS-stimulated RAW 264.7 cells. These inhibitory effects were attributable to the prevention of I*κ*B-*α* degradation, thereby suppressing NF-*κ*B activation. Although the exact mechanisms regulating the anti-inflammatory activity of sargachromenol are not yet fully known, our findings suggest that sargachromenol may have the potential to prevent inflammatory diseases and may act as a modulator of macrophage activation. Future studies are expected to confirm the anti-inflammatory effects of sargachromenol in a representative anti-inflammatory animal model.
